# Right Ventricular Perforation with the Body of Swan-Ganz Catheter during Lung Transplantation by ECMO Support: A Case Report

**Published:** 2017

**Authors:** Alireza Jahangirifard, Zargham Hossein Ahmadi, Ali Khalili, Abolghasem Daneshvar Kakhaki, Kambiz Sheikhy

**Affiliations:** 1 Lung Transplantation Research Center, National Research Institute of Tuberculosis and Lung Diseases (NRITLD), Shahid Beheshti University of Medical Sciences, Tehran, Iran.,; 2 Tracheal Diseases Research Center, NRITLD, Shahid Beheshti University of Medical Sciences, Tehran, Iran

**Keywords:** Right ventricle, Perforation, Lung transplantation, Extracorporeal Membrane Oxygenation (ECMO)

## Abstract

A 46-year-old woman with a 12-year history of lymphangiomyomatosis (LAM) was admitted for lung transplantation in January 2017. We decided to apply veno-arterial extracorporeal membrane oxygenation (ECMO) to manage arrhythmia and hypotension during lung transplantation, since it was not controllable with inotropic drugs. After transplanting the right (first) lung and at the time of left pneumonectomy, the body of the Swan-Ganz catheter was suddenly observed to be protruding from the right ventricular (RV) wall. The catheter was found folded at part of its body and ran out 0.5 cm from the RV. The protruding part of the catheter was inserted before the perforated part of the cardiac muscle was repaired in order to control the bleeding. ECMO was used throughout the rest of the procedure and the patient was transferred to the intensive care unit (ICU) in good condition before being weaned from the ventilator after 16 hours. It seems that gentle manipulation, concurrent use of transesophageal echocardiography (TEE), insertion of the appropriate length of the catheter into the heart chambers, and a softer material in the structure of the catheters would be helpful to prevent these kinds of potentially fatal complications.

## INTRODUCTION

Transcardiac catheterization was first performed in 1929 by Dr. Forssmann, who passed a catheter through his own heart. The Swan-Ganz catheter was introduced later, in 1970, as a type of pulmonary artery catheter to diagnose hemodynamic abnormalities in the heart and lung, in order to determine the feasibility of right heart catheterization (1, 2). Heart failure, post-attack heart dysfunction, pulmonary edema, congenital heart disease, postoperative monitoring of open heart surgery, valvular heart diseases, cardiomyopathy, and pulmonary hypertension are the main fields in which the Swan-Ganz catheter is being used (2).

Bilateral lung transplantation (BLTX) is a globally accepted treatment for many end-stage pulmonary diseases. Increasing pulmonary artery pressure is a reasonable indication for applying extracorporeal membrane oxygenation (ECMO) at the time of lung transplantation, and may help the anesthetist in monitoring and managing hemodynamic instability during the procedure. Early postoperative morbidity and mortality, mainly due to left or right ventricular dysfunction, are reasonable indications to use postoperative extracorporeal membrane oxygenation (ECMO) as a weaning strategy after lung and heart surgeries (3). Despite its wide range of applications, Swan-Ganz catheterization is associated with complications, even death; so, it needs to be utilized by experienced operators, although some complications are independent of any profession or experience.

## CASE SUMMARIES

**Figure 1.**
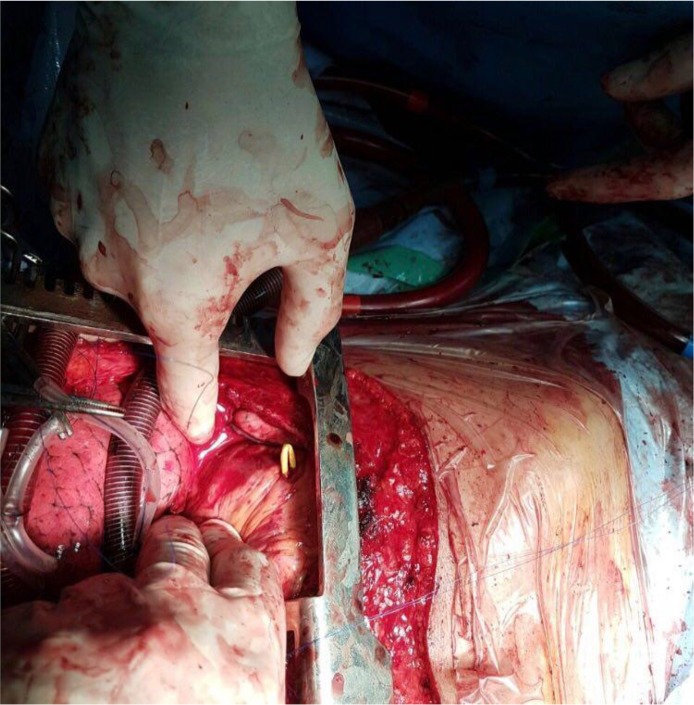
The protruded part of the body of the Swan-Ganz catheter is clearly observable extruding the right ventricle wall

A 46-year-old woman was referred to our center with a 12-year history of lymphangiomyomatosis (LAM). She has been a candidate for lung transplantation since 2007. She complained of severe dyspnea and was function class III of the American Heart Association classification. Her past medical history showed no other co-morbidities, and Seroflo (Salmeterol + Fluticasone) and Atrovent (Ipratropium bromide) sprays were the only drugs she had used. On physical examination, pulse rate was 99/minute, respiratory rate was 20/minute, and blood pressure was 100/60 mmHg. There were diminished pulmonary sounds on chest auscultation. On echocardiography, left ventricular ejection fraction was 55%, right ventricle (RV) size was normal, and there was mild dysfunction of the RV. There was no abnormality in her coagulation profile. She was admitted for lung transplantation in January 2017. Five-lead ECG monitoring was started after preparation, along with pulse oximetry and cerebral oximetry monitoring, before setting up the arterial line. The Swan-Ganz catheter was inserted from the right internal jugular vein after anesthesia induction was completed, and passed through the right chambers with no resistance; it was continuously used for wave monitoring before stopping at the beginning of the pulmonary artery. The pulmonary artery pressure (PAP) was 38/21 mmHg, and was continuously monitored throughout the operation. Central veno-arterial extracorporeal membrane oxygenation (V-A ECMO) was applied when intractable arrhythmia and hypotension occurred during the first pneumonectomy (right). ECMO canulation was performed through right atrium using a two stage venous cannula and aorta using a 24 Fr. canulla (Medtronic, Inc., Minneapolis, MN, USA). Shortly after the left pneumonectomy was started, the body of the Swan-Ganz catheter was suddenly observed to be protruding from a small opening on the wall of the RV, which a yellowish wire was partially protruded. Upon closer examination (Figure 1), the catheter was found folded over itself and ran out 0.5 cm from the RV wall. The protruding part of the catheter was re-inserted into the RV before suturing the perforated part of the cardiac muscle in order to control the bleeding. The catheter tip was positioned at the pulmonary artery, and this was double checked by the surgeon manually, in addition to the wave monitoring. ECMO was used throughout the rest of the procedure and the patient was transferred to the intensive care unit (ICU) in good condition, before being weaned from the ventilator after 16 hours.

## DISCUSSION

The Swan-Ganz balloon flotation catheter has several advantages, among which simple usage is the most popular, because it needs neither vast experience nor professional techniques like fluoroscopy for placement at bedside, even in critically ill patients (1).

With regard to complications, the Swan-Ganz catheter has been associated with rare reports of pulmonary artery (PA) pseudoaneurysm due to PA perforation during insertion, which is a fatal event. Poplausky et al. described three cases of this complication in 3 women with different backgrounds and with different pseudoaneurysm locations in the pulmonary circulation (4). Boyd et al. found that the rate of PA rupture was 0.2% among 528 patients undergoing Swan-Ganz placement. The total complications associated with catheter insertion have been estimated to be less than 20% by several authors (4–7).

In 2008, de Jesus Peixoto Camargo et al. reported a catheter knot when they were inserting a Swan-Ganz catheter on the right side to monitor a 52-year-old man with severe emphysema who was a candidate for right lung transplantation (8). This complication has been repeatedly reported due to the wide usage of the Swan-Ganz catheter since 1970. Ventricular perforation is a relatively rare complication, and has been reported with a frequency of 0.1% (8). Bossert et al. revealed 4 complications of the catheter in 4 separate cases; among them was a 64-year-old man with acute myocardial infarction (MI) who underwent coronary artery bypass grafting (CABG). A Swan-Ganz catheter was inserted through the right internal jugular vein and was used to monitor his hemodynamic status (9). The patient developed cardiac tamponade and needed to undergo a median sternotomy for exploration, wherein a right ventricular perforation was noted and subsequently controlled with pericardial patch-supported closure. The aforementioned study stated that advanced age (>60 years), female sex, pulmonary hypertension, coagulation disorders and anticoagulation therapy, hypothermia, intraoperative heart manipulation, and catheter rigidity are the main risk factors for the development of general complications of Swan-Ganz catheter insertion. Some sources also consider chronic steroid administration as a risk factor, due to increased vascular system fragility (4, 9, 10). Bossert et al. divide the serious complications of catheterization into 3 categories: associated with insertion and installation, indwelling, and finally, removal. Right ventricular perforation may occur during Swan-Ganz catheterization or pacemaker placement, resulting in cardiac tamponade, but is generally a rare occurrence (9, 11–14).

A case of right ventricular perforation due to Swan-Ganz catheter insertion in a 71-year-old man during CABG surgery was also reported by Kim et al. in 2016 (15). His echocardiography showed an ejection fraction (EF) of 61%, there was no history of MI, and vital signs were stable. No resistance or difficulty was reported during the insertion and installation phase. A sudden increase in the diastolic pressure confirmed proper placement of the catheter in the PA. Suddenly, protrusion of the tip of the catheter from the RV was discovered when the team was manipulating the heart for anastomosis of the greater saphenous vein and the posterolateral branch of the right coronary artery. The catheter was removed completely before closing the perforated site with 4-0 Prolene (polypropylene) sutures and Bioglue, and the CABG was completed with no other events. The author addressed the length of the needed catheter for different races, as they noted that longer measures were needed for Western people while shorter lengths were needed in Indian patients. Sufficient withdrawal of the catheter to the right atrium may be different from case to case, and there is no strict rule in this regard; this is the point where more experience may play a crucial role. More residual length of the catheter in the RV seems to be a risk factor for RV perforation, especially during heart manipulation (15).

In our case, it is worth noting that the tip of the catheter was not the problem; rather, it was the body that perforated the RV. This is the first report, to our knowledge, wherein the body of the Swan-Ganz catheter protruded from the cardiac wall, which was directly associated with manipulation and excessive length of the catheter inserted into the heart chambers. The protrusion of the body of the Swan-Ganz catheter was a completely new challenge that we had not anticipated. Manipulation of the heart made the event more likely to occur, and as some authors pointed out, the rigidity of the catheter may be a risk factor for perforation. The current reported patient had no risk factor for a vulnerable cardiac or vascular wall, such as a prior MI, chronic steroid administration, or even anticoagulation (16–18). We did not use transesophageal echocardiography (TEE) during our procedure to confirm the position of the catheter, but some experts suggest its use to prevent or at least lessen catheter-induced heart injuries (15). The majority of intravascular catheters are made from very soft materials; the Swan-Ganz catheter; however, is made from stiff material to lessen the rate of knotting or formation of sharp curves during installation. This is also a risk factor for rupture (15, 19), especially with its tip, although we believe our case was due to body protrusion instead of the tip.

To conclude, gentle manipulation, concurrent use of TEE, prevention of insertion of an excessive length of the catheter into the heart chambers, and finally, a softer material in the structure of catheters would be helpful to prevent these kinds of potentially fatal complications.
